# Automatic diagnosis of pediatric supracondylar humerus fractures using radiomics-based machine learning

**DOI:** 10.1097/MD.0000000000038503

**Published:** 2024-06-07

**Authors:** Wuyi Yao, Yu Wang, Xiaobin Zhao, Man He, Qian Wang, Hanjie Liu, Jingxin Zhao

**Affiliations:** a Department of Orthopedics, Affiliated Hospital of Chengde Medical University, Chengde, Hebei, PR China; b Department of Radiology, Affiliated Hospital of Chengde Medical University, Chengde, Hebei, PR China; c Department of Rehabilitation, Affiliated Hospital of Chengde Medical University, Chengde, Hebei, PR China; d Department of Orthopedics, Tianjin Beichen Hospital, Tianjin, PR China.

**Keywords:** artificial intelligence, diagnosis, machine learning, radiomics, supracondylar fracture

## Abstract

The aim of this study was to construct a classification model for the automatic diagnosis of pediatric supracondylar humerus fractures using radiomics-based machine learning. We retrospectively collected elbow joint Radiographs of children aged 3 to 14 years and manually delineated regions of interest (ROI) using ITK-SNAP. Radiomics features were extracted using pyradiomics, a python-based feature extraction tool. T-tests and the least absolute shrinkage and selection operator (LASSO) algorithm were used to further select the most valuable radiomics features. A logistic regression (LR) model was trained, with an 8:2 split into training and testing sets, and 5-fold cross-validation was performed on the training set. The diagnostic performance of the model was evaluated using receiver operating characteristic curves (ROC) on the testing set. A total of 411 fracture samples and 190 normal samples were included. 1561 features were extracted from each ROI. After dimensionality reduction screening, 40 and 94 features with the most diagnostic value were selected for further classification modeling in anteroposterior and lateral elbow radiographs. The area under the curve (AUC) of anteroposterior and lateral elbow radiographs is 0.65 and 0.72. Radiomics can extract and select the most valuable features from a large number of image features. Supervised machine-learning models built using these features can be used for the diagnosis of pediatric supracondylar humerus fractures.

## 1. Introduction

Supracondylar fracture of humerus refers to a fracture of 2 to 3 cm above the internal and external condyles of humerus, and is the most common type of elbow fracture in children, accounting for 3% of all pediatric fractures.^[1]^ Due to the special anatomy of the weak intercondylar fossa connecting the supracondylar region of the humerus, children are prone to fractures when they fall.^[2]^ Supracondylar humerus fractures often lead to catastrophic complications such as nerve and vascular damage, compartment syndrome, and elbow joint deformities.^[3]^ Therefore, accurate diagnosis and proper management are crucial.

Standard anteroposterior and lateral elbow radiographs are essential for the diagnosis of supracondylar fractures of the humerus. Most supracondylar fractures show obvious fracture lines and displacement on radiographs. However, some occulted fractures are not apparent on radiographs, and due to the presence of ossification centers that change with age,^[4]^ misdiagnosis and missed diagnosis may occur during diagnosis, especially when the attending physician is not a specialized pediatric orthopedist.

Artificial intelligence can extract and screen information useful for diagnosis and treatment from medical data, especially in the field of recognition of medical image data has made great breakthroughs.^[5]^ Medical artificial intelligence assists the work of clinicians to provide accurate and optimized medical services for patients, which is an inevitable trend of future medicine. Artificial intelligence technologies, including deep learning, radiomics, and radiomics-based machine learning, are valuable for clinical diagnosis and treatment in medical practice.^[6,7]^ Radiomics is a rapidly developing research field in recent years. It uses computer methods to extract a large amount of high-dimensional data from image images and capture and quantify image features that cannot be recognized by human eyes, after feature calculation and selection, reduction and data processing, the disease characteristics are comprehensively quantified through the development and utilization of algorithms such as machine learning and deep learning.^[8]^ Radiomics has advantages over human brain in processing large amounts of data and multidimensional data. The aim of this study was to develop a supervised machine-learning model for the automatic diagnosis of pediatric supracondylar humerus fractures on conventional radiography.

## 2. Materials and methods

### 2.1. Clinical data

**Figure 1.**
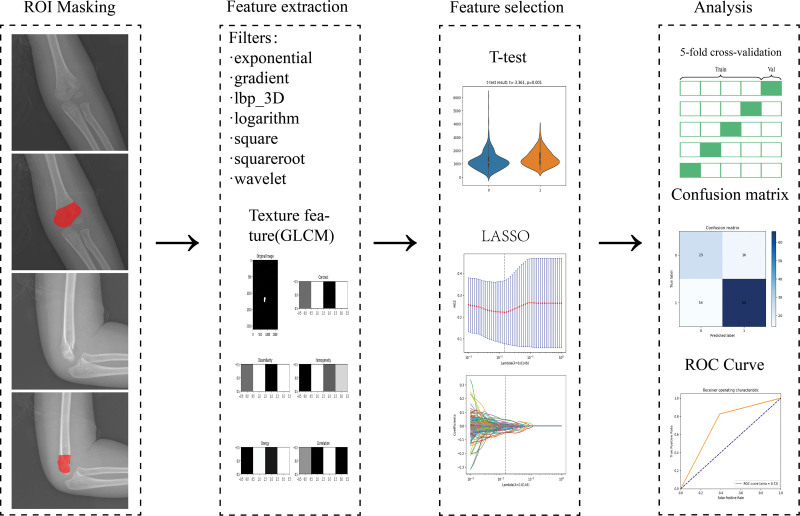
Flowchart of the study. The method of manually separating regions of interest (ROI) was adopted. Firstly, the radiomic features are extracted from the mask map, the filters and texture features using a Gray Level Co-occurrence Matrix (GLCM) as an example are presented. Next, a feature selection step is performed on the extracted features using the T-test and the least absolute shrinkage and selection operator (LASSO). Thereafter, Perform 5-fold cross-validation in the training set. Finally, confusion matrix and ROC curve were used to evaluate the diagnostic efficiency of the model.

In this study, standard anteroposterior and lateral elbow radiographs of children aged 3 to 14 years were derived from the PACS system of the Affiliated Hospital of Chengde Medical College. A supracondylar fracture of the humerus is diagnosed when any of the following criteria are met: obvious supracondylar fracture line, anterior humeral line passing more anterior to the capitellum, buckling of the cortex, positive anterior fat pad sign (sail sign), or positive posterior fat pad sign. Additionally, X-rays of normal elbow joints were selected at a ratio of 2:1 for inclusion in the study. the data analysis flowchart of the study is shown in Figure 1.

### 2.2. Segmentation of ROI

In this study, the method of manually separating regions of interest (ROI) was adopted, and the operations of delineating ROI were as follows: First, the images were imported into ITK-SNAP (version: 3.8.0), and the contour of each humeral condyle on the anteroposterior and lateral elbow radiographs was manually sketched to generate an ROI mask on the images. This work was jointly completed by 2 radiologists and pediatric orthopedic surgeons with many years of experience, and the results were reviewed by a senior physician who has been engaged in pediatric orthopedic work for more than 10 years. Inconsistent judgments need to be unified through discussion.

### 2.3. Feature extraction

The method of feature extraction in this study is to save DICOM file of radiographs with outlined ROI as NIFTI format, and use pyradiomics package to extract imaging features in corresponding image positions based on each ROI mask in Python (v3.7.0) language environment. We used 7 types of filters as follows: exponential, gradient, lbp_3D, logarithm, square, squareroot, and wavelet (wavelet-HHH; wavelet-HHL; wavelet-HLH; wavelet-HLL; wavelet-LHH; wavelet-LHL; wavelet-LLH; wavelet-LLL). And contains 7 radiomic class: First Order Statistics, shape, Gray Level Co-occurrence Matrix (glcm), Gray Level Run Length Matrix (glrlm), Gray Level Size Zone Matrix (glszm), Gray Level Dependence Matrix (gldm), and Neighboring Gray Tone Difference Matrix (ngtdm).

### 2.4. Feature selection

The performance of the model is better when the data characteristics conforming to the standard normal distribution are used. Therefore, in feature selection in this study, the scipy package t-test in python (version number: 3.7.0) was first used to conduct difference significance test between groups (t < 0.05), so that all features with non-zero coefficients could be obtained for subsequent feature screening. After that, the LASSO was used to screen the features. The error value of cross-validation was 10, and the maximum number of iterations was 100000.

### 2.5. Classification model and evaluation

Based on the selected features, several supervised learning models are available for classification analysis. In this study, the radiomics-based models were constructed with LR. The data should be split into a training set and a test set in an 8:2 ratio. The hyperparameters of the LR classifier, including penalty, C, and solver, should be tuned in the training set. The stability of the model should be evaluated through 5-fold cross-validation in the training set. Finally, the LR model with the optimized hyperparameters should be applied to the test set. The diagnostic performance of the model should be evaluated using ROC curve and area under the curve (AUC). In addition, the evaluation index data also include, Accuracy = (True Positive + True Negative)/ (True Positive + True Negative + False Positive + False Negative), Precision = True Positive/ (True Positive + False Positive), Recall = True Positive/(True Positive + False Negative), Specificity = True Negative/(True Negative + True Negative) and F1-Score = PPV × TPR × 2/(PPV + TPR). The classification of the test set is represented in the form of a prediction probability graph with classification error marks.

## 3. Result

### 3.1. Clinical data selection results

A total of 411 fracture samples and 190 normal samples were included in the study. The ratio of fracture sample data to normal data is close to 2:1, so there is no need to perform sample balancing.

### 3.2. Radiographs feature extraction and screening

**Figure 2.**
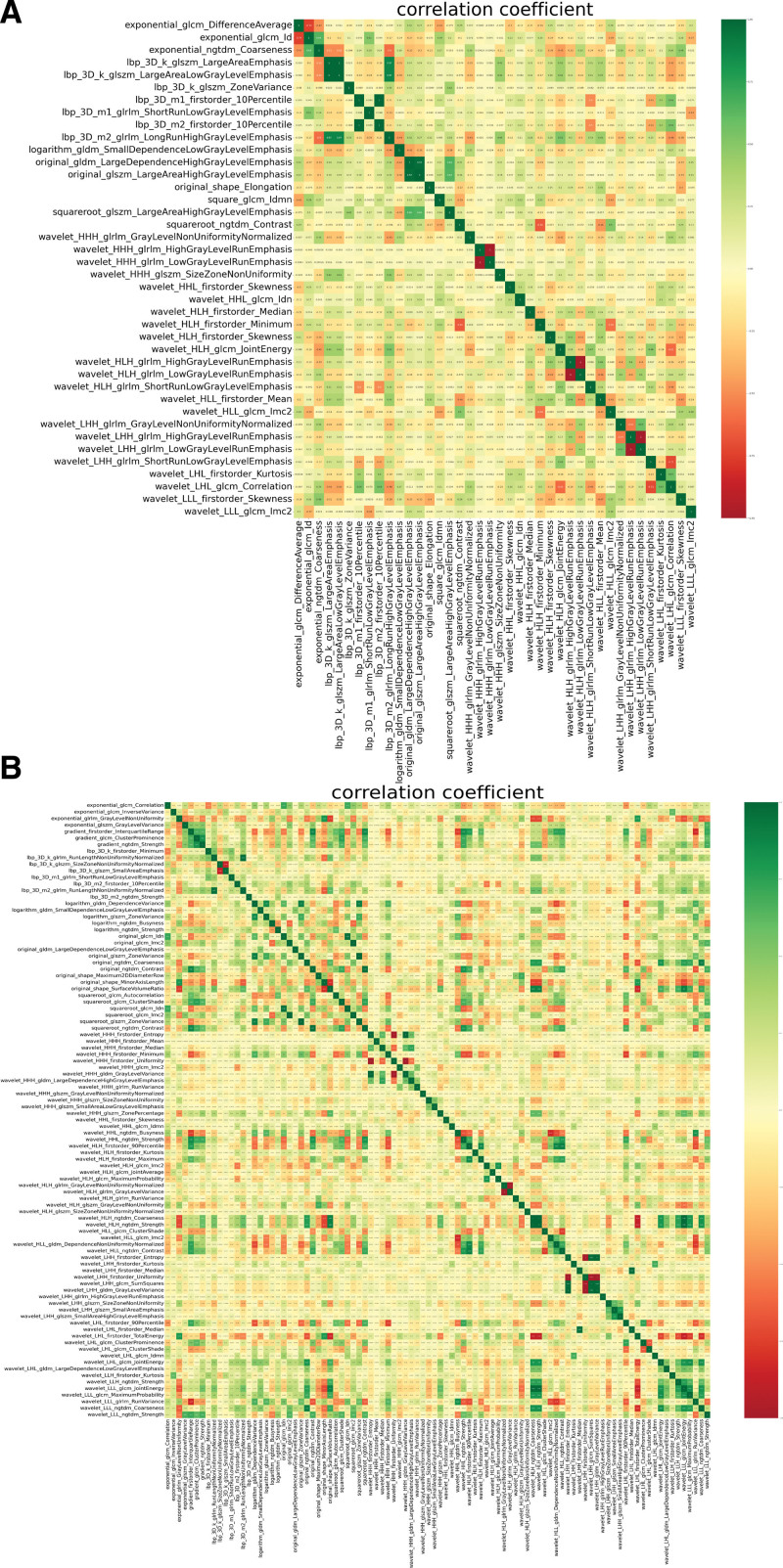
Heat map of correlation coefficient between features. (A) Heat map of correlation coefficient between features on the anteroposterior (B) Heat map of correlation coefficient between features on the lateral elbow radiographs. The darker the color in the figure, the higher the correlation.

**Figure 3.**
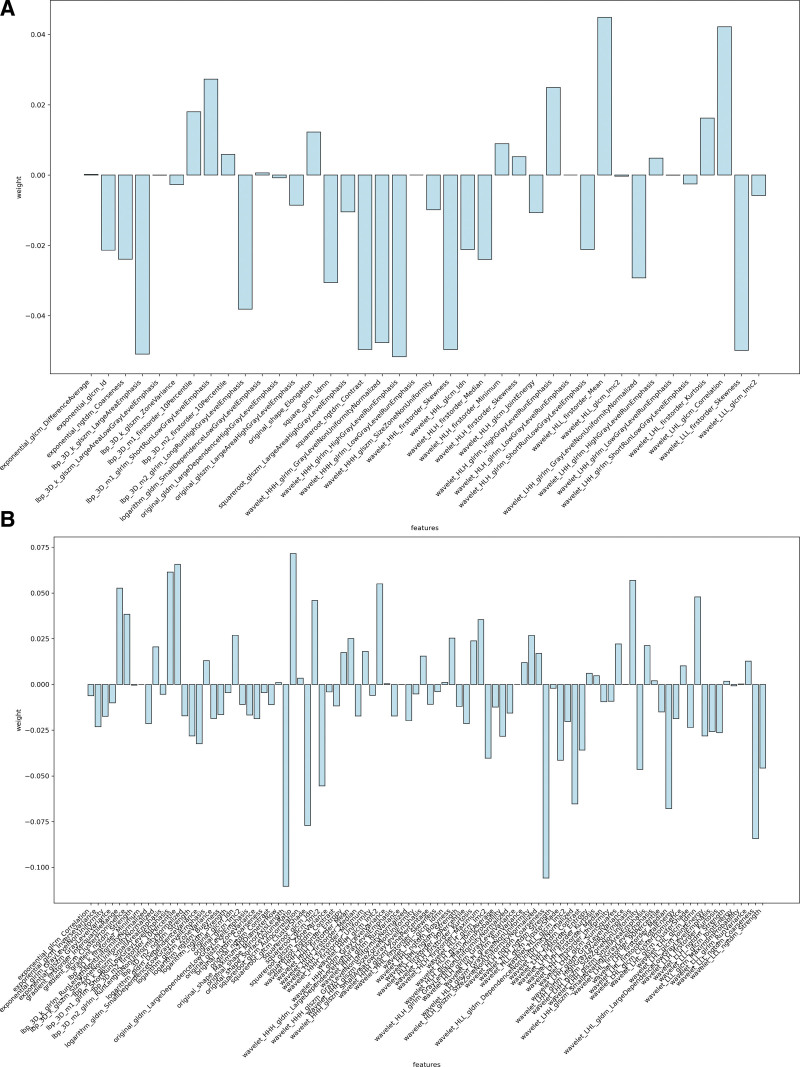
Feature weight graph. (A) The feature weight map of the anteroposterior; (B) Correlation coefficient between 94 features on the lateral elbow radiographs. The horizontal coordinate is the name of each feature, The absolute value of the ordinate represents its specific gravity.

For each ROI, a total of 1561 features were extracted for subsequent analysis. These characteristics were divided into 3 groups: Group 1 (First Order Statistics), included First Order; Group 2 (shape- and size-based features), included Shape; Group 3 (texture features), included glcm, glrlm, glszm, gldm and ngtdm. After t-test and feature screening by LASSO, 40 features with the most diagnostic value were finally selected for further classification modeling on the forward slice, while 94 features were selected for classification modeling on the lateral slice. Figure 2 shows the name of each feature and the correlation coefficient between each feature. The weight of each feature is shown in Figure 3.

**Figure 4.**
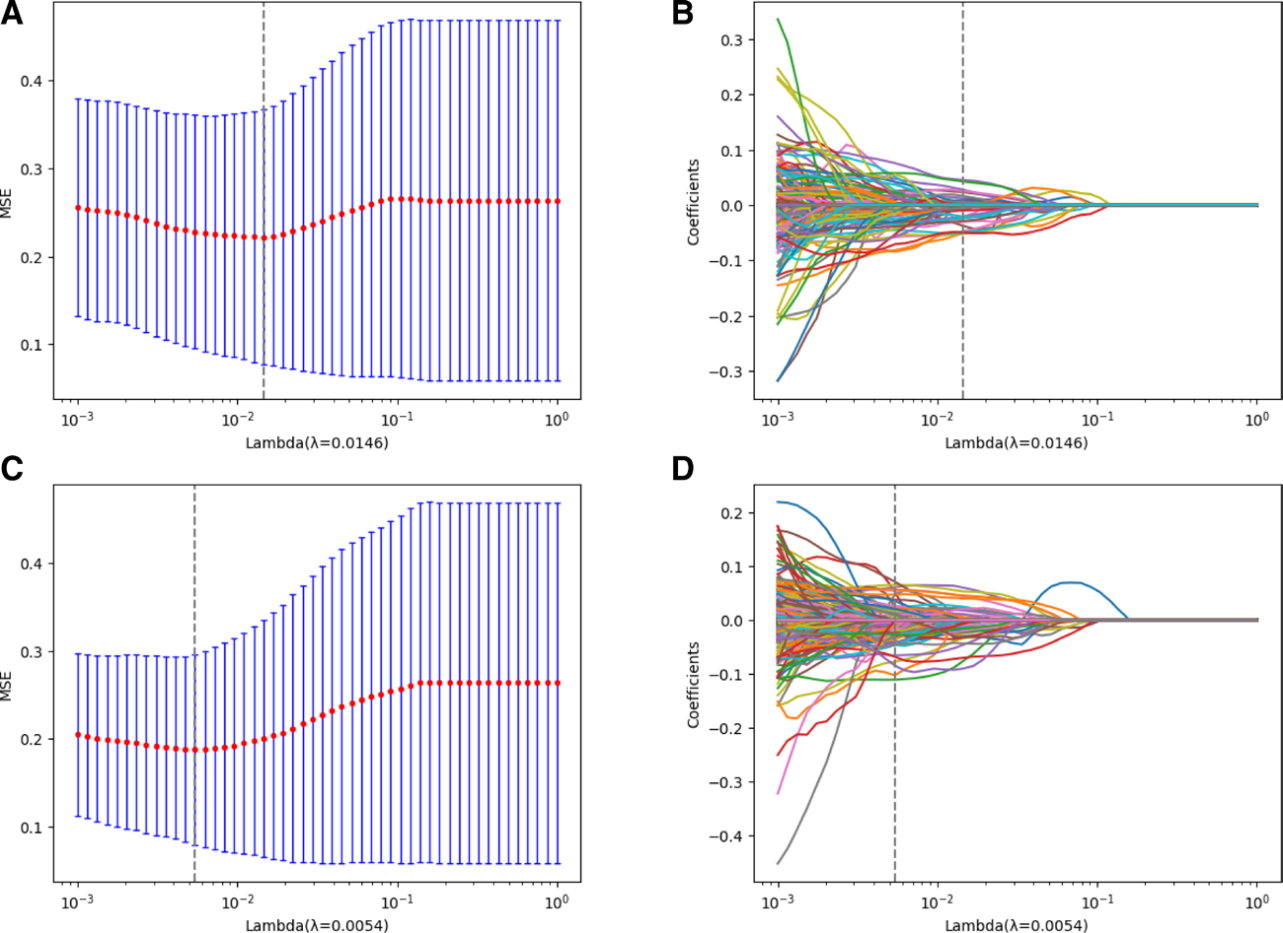
LASSO regression screening of radiomics features. (A, C) LASSO adjusts different parameters (λ) through 10-fold cross-validation to minimize Mean Square Error, so as to screen out the feature set with the best performance; (B, D) Graph showing that the coefficients of radiomics vary with the parameters (λ). LASSO = the least absolute shrinkage and selection operator.

After feature screening by LASSO regression algorithm, the best λ value is selected on the front and side slices respectively, and the model established under this λ value has the smallest error and the best diagnostic effect. The best λ value obtained on the anteroposterior is 0.0146 (Fig. 4A–B). The best λ value on the lateral elbow radiographs was 0.0054 (Fig. 4C–D).

### 3.3. LR model adjusts hyperparameters

The grid search in the GridSearchCV package in python is used to adjust hyperparameters, penalty is selected from l1 and l2, and the value of C ranges from 0.01, 0.1, 1,10. solver includes 4 solvers: liblinear, sag, newton-cg, and lbfgs. The method of 10-fold cross-validation was used to find the best combination of hyperparameters. The final combination of hyperparameters on the anteroposterior slice was as follows: C = 1, penalty = l1, solver = liblinear; The combination of hyperparameters on the lateral elbow radiographs is C = 10, penalty = l1, solver = liblinear.

### 3.4. Evaluation of diagnostic performance of the model

**Figure 5.**
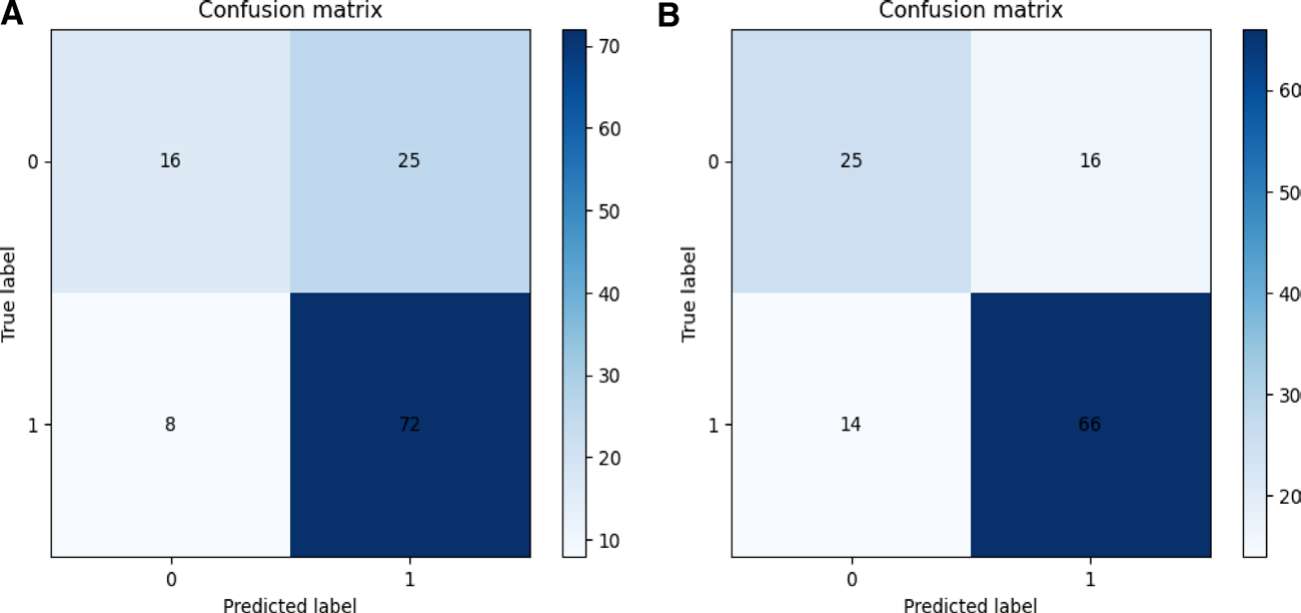
Confusion matrix. (A) visual confusion matrices of the anteroposterior; (B) visual confusion matrices of the lateral elbow radiograph. The horizontal coordinate is the predicted value, and the vertical coordinate is the true label. 1 represents a fracture sample, 0 represents a normal sample.

**Figure 6.**
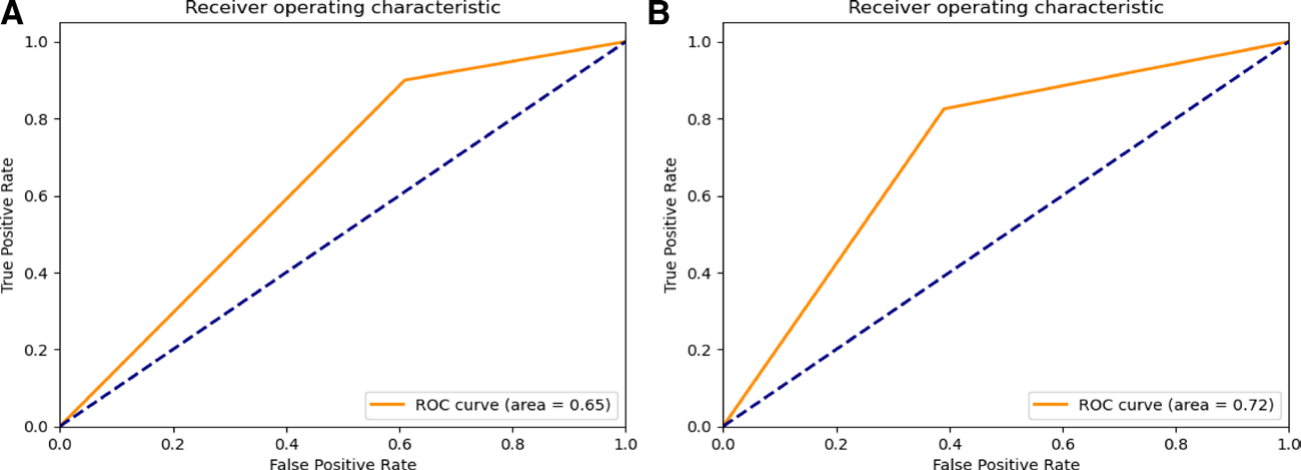
ROC curves of the logistic regression model. (A) ROC curve of the anteroposterior; (B) ROC curve of the lateral elbow radiograph. ROC = receiver operating characteristic.

The 5-fold cross-validation was performed in the training set, and the results were recorded together with the test set in Table 1. In the test set, the AUC of the LR model was 0.65 and 0.72 on the anteroposterior and lateral elbow radiographs. Figure 5 for visual confusion matrix and Figure 6 for ROC curve.

**Table 1:** Evaluation indexes of 5-fold cross-validation and test set.

	Accuracy	Specificity	Sensitivity	Precision	AUC	F1-score
0-fold Anteroposterior	0.760	0.430	0.909	0.780	0.669	0.840
Lateral elbow radiographs	0.869	0.732	0.931	0.885	0.831	0.907
1-fold Anteroposterior	0.756	0.416	0.909	0.776	0.663	0.837
Lateral elbow radiographs	0.869	0.725	0.934	0.883	0.829	0.907
2-fold Anteroposterior	0.763	0.436	0.909	0.782	0.673	0.841
Lateral elbow radiographs	0.869	0.725	0.934	0.883	0.829	0.907
3-fold Anteroposterior	0.763	0.436	0.909	0.782	0.673	0.841
Lateral elbow radiographs	0.869	0.732	0.931	0.885	0.831	0.907
4-fold Anteroposterior	0.763	0.436	0.909	0.782	0.673	0.841
Lateral elbow radiographs	0.869	0.732	0.931	0.885	0.831	0.907
Test set Anteroposterior	0.727	0.390	0.900	0.742	0.645	0.814
Lateral elbow radiographs	0.760	0.610	0.838	0.807	0.724	0.822

AUC = area under curve.

### 3.5. Prediction probability graph of test set

**Figure 7.**
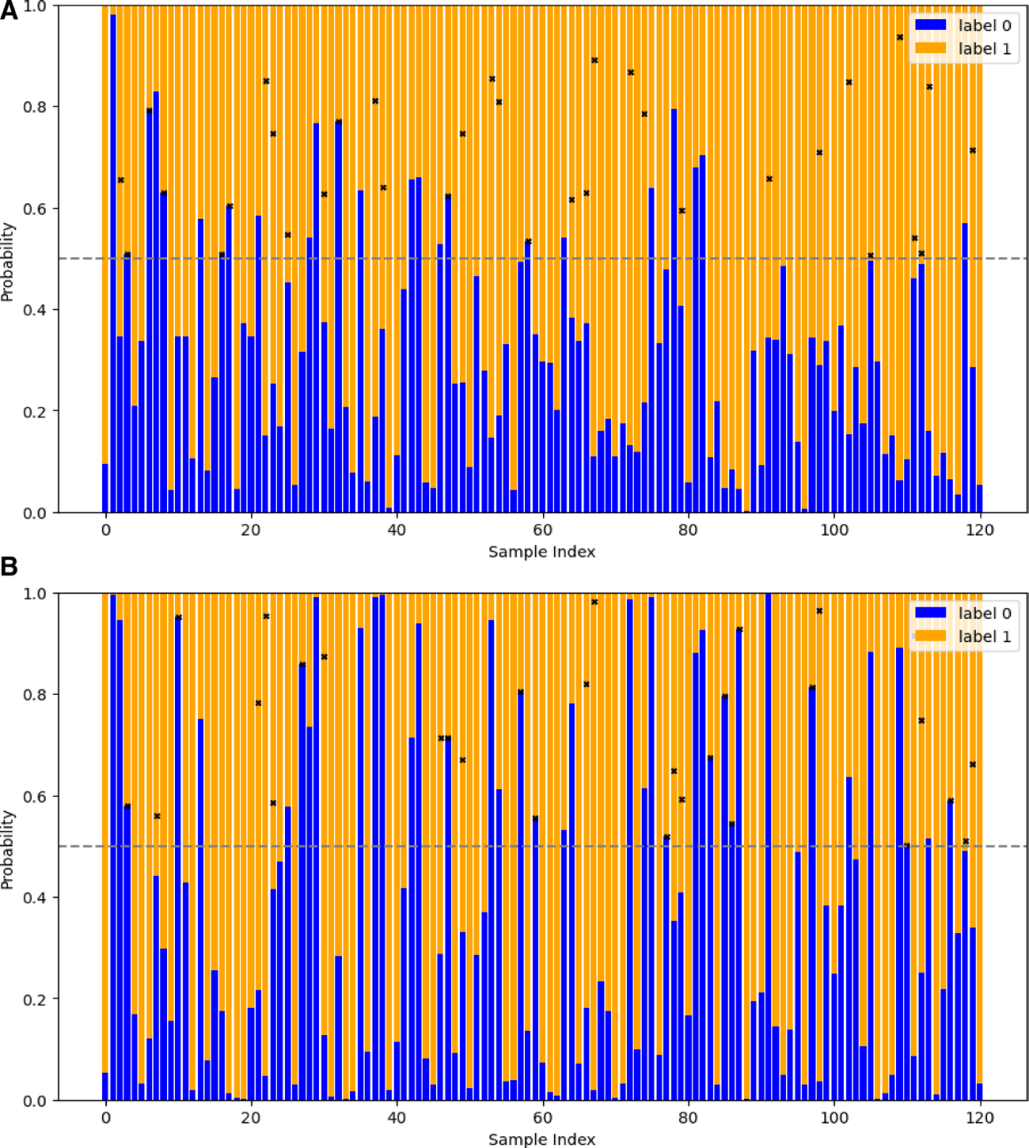
Prediction probability graph of test set. (A) prediction probability graph of the anteroposterior; (B) prediction probability graph of the lateral elbow radiographs. The horizontal coordinate is for each sample in the test set, the ordinate is the probability that the sample is predicted to be 0 or 1 by the logistic regression model, the dashed line is the threshold (0.5).

The Prediction probability graph (Fig. 7) can intuitively show the classification of each sample in the test set, the sample marked with a black cross represents the sample of the model classification error. The threshold means that the probability of predicting a label is >0.5, that is, the sample is predicted to be of this class.

## 4. Discussion

The main work of this study is to extract and screen out the features with diagnostic value in radiographs, and to use these features to establish a machine-learning model that can predict the diagnosis of pediatric supracondylar humerus fractures. Our results show that the LR model has good performance in diagnosing pediatric supracondylar humerus fractures.

Artificial intelligence technologies, including deep learning, radiomics, and radiomic-based machine learning, have been prominent in the field of medicine.^[9,10]^ Most of the previous studies focused on the research field of adult orthopedics, and few literatures reported the application of radiomics artificial intelligence in pediatric orthopedics. Abhinav Suri and Brandon C Jones developed a neural network that can segment vertebra and disc in MR, CT and X-ray quickly and accurately.^[11]^ Chiari-Correia NS train an artificial neural network model using 3D radiomic features to differentiate benign from malignant vertebral compression fractures (VCFs) on MRI.^[12]^ According to Jin research report,^[13]^ they obtained good accuracy in distinguishing injury time of rib fracture by using radiomic-based machine learning. Üreten developed a computer-aided diagnosis (CAD) method to assist physicians in the diagnosis of hand fractures using artificial intelligence methods.^[14]^ Hong N study found that the bone radiomics score derived from texture features of DXA hip images improved hip fracture risk prediction in community-dwelling older women.^[15]^ Asma Alzaid used artificial intelligence to automate the detection and classification of peri-prosthetic femur fracture.^[16]^ Choi JW developed a deep learning neural network that can automatically identify pediatric supracondylar humerus fractures,^[17]^ which is one of the early studies on the application of artificial intelligence in pediatric orthopedics. Michel Dupuis research confirmed that artificial intelligence very reliable for detecting fractures in children, especially in those older than 4 years and without cast.^[18]^ At the same time, Eszter Nagy present a comprehensively annotated pediatric wrist trauma radiography dataset for machine learning,^[19]^ which provides convenience for the follow-up evaluation of children wrist injuries. However, few reports have applied such machine-learning techniques to the diagnosis of pediatric supracondylar humerus fractures.

It can be seen from Figure 2 that most of the image omics features finally used for modeling are based on texture features. This phenomenon is expected, because texture is a visual feature that reflects the homogeneity of the image, it reflects the surface structure of the object with slow or periodic changes in the organization of the property, and is usually the best performance in the radiomic features.^[20,21]^ In this study, we performed supervised machine learning using a logistic regression model, and our results show that machine learning can learn certain radiomic features that can be used to diagnose pediatric supracondylar humerus fractures on radiographs. In the machine-learning model, the performance on the lateral radiographs was better than that on the anterior radiographs, which may be explained by the presence of more imaging features distinguishing fractures on the lateral radiographs. In addition, it can also be seen from the prediction probability chart that the prediction probability of the sample with the wrong judgment of the model is relatively low. In the training set, we have adjusted the LR model very stable, although the performance in the test set is not relatively perfect, but AUC > 0.65 indicates that our research is successful.

Limitations of this study: First, the sample size of this study is small, which needs to be further expanded. Second, this retrospective study only proves that imaging artificial intelligence can be used as an auxiliary tool to diagnose supracondylar fracture of humerus, and prospective studies can be carried out in the future to help doctors in clinical work. Third, compared to the training set, the results of the test set are not so perfect, and we can further optimize the model to get more satisfactory results.

## 5. Conclusions

The imaging features extracted and screened from the radiographs are valuable for the identification of pediatric supracondylar humerus fractures, and the machine-learning model established by using these features can make a more accurate diagnosis of supracondylar fractures in children. Radiomics-Based Machine Learning on Radiographs in Pediatric supracondylar humerus fractures has tremendous potential, and it is expected to be applied in clinical work.

## Acknowledgments

We thank all of the patients involved in the study.

## Author contributions

**Investigation:** Man He, Hanjie Liu.

**Software:** Qian Wang.

**Resources:** Yu Wang.

**Visualization:** Xiaobin Zhao.

**Writing – original draft:** Wuyi Yao.

**Writing – review & editing:** Yu Wang, Jingxin Zhao.
